# Stochastic mortality forecasts for Bangladesh

**DOI:** 10.1371/journal.pone.0276966

**Published:** 2022-11-10

**Authors:** Ahbab Mohammad Fazle Rabbi, Hafiz T. A. Khan

**Affiliations:** 1 Department of Population Sciences, University of Dhaka, Dhaka, Bangladesh; 2 Professor of Public Health & Statistics, University of West London, London, United Kingdom; 3 Associate Professorial Fellow, The Oxford Institute of Population Ageing, The University of Oxford, Oxford, United Kingdom; Oswaldo Cruz Foundation, BRAZIL

## Abstract

Mortality forecasts are essential part for policymaking in any aging society. In recent years, methods to model and forecast mortality have improved considerably. Among them, Lee-Carter method is one of the most influential method. In this paper, Lee-Carter method is applied to forecast mortality and life expectancy of Bangladesh. A functional data analysis approach is used to decompose the smoothed log-mortality rates in Lee-Carter framework for higher goodness-of-fit of the models and for longer forecast horizons. Bangladesh has been experiencing a mortality transition and has gained life expectancy in last few decades. The fitted model here showed higher pace of mortality decline for women in Bangladesh than that of men. The forecasts showed continuation of mortality improvement in long run and by 2060 life expectancy at birth is expected to reach over 80 years for both sexes in Bangladesh. The study also predicts the effect of reduction in infant mortality on the life expectancy in Bangladesh.

## Introduction

In the last century, the human mortality has declined globally except for some certain irregularities [1–3]. This improvement is an outcome of modern health-care systems, awareness of people regarding health behavior. The declining trend in mortality help us to accurately forecast life expectancy as a core requirement for decision-making in social, healthcare and financial sectors. Fundamental changes of welfare policies largely depend on the accurate forecast of longevity in any country. Stochastic modeling of mortality forecasting is gaining popularity in this context; United Nations and several industrialized countries already adapted stochastic forecasting techniques [4, 5]. Several probabilistic approaches exist for mortality forecasting, both from Frequentist and Bayesian point of view. Among several different approaches of stochastic mortality forecasting, undoubtedly the most prominent method till now is the Lee-Carter method [6]. The forecasts of the various life-table functions obtained from this method have probability distributions, so probability intervals can be calculated for each variable and for summary measures such as life expectancy. This method decomposes the differences of log-mortality rates and average mortality level into two parts: an invariant age component and a time component. Forecasting is done by standard time series forecasting on time component, considering same structure of age-specific mortality level over time. Later, several other modifications were proposed on the basic method and huge literature exist on application of these methods to forecast mortality and life expectancies for low-mortality countries. It should be noted that, all these method work well for low-mortality regime where almost steady decline in age-specific mortality rates are observed.

**Fig 1:**
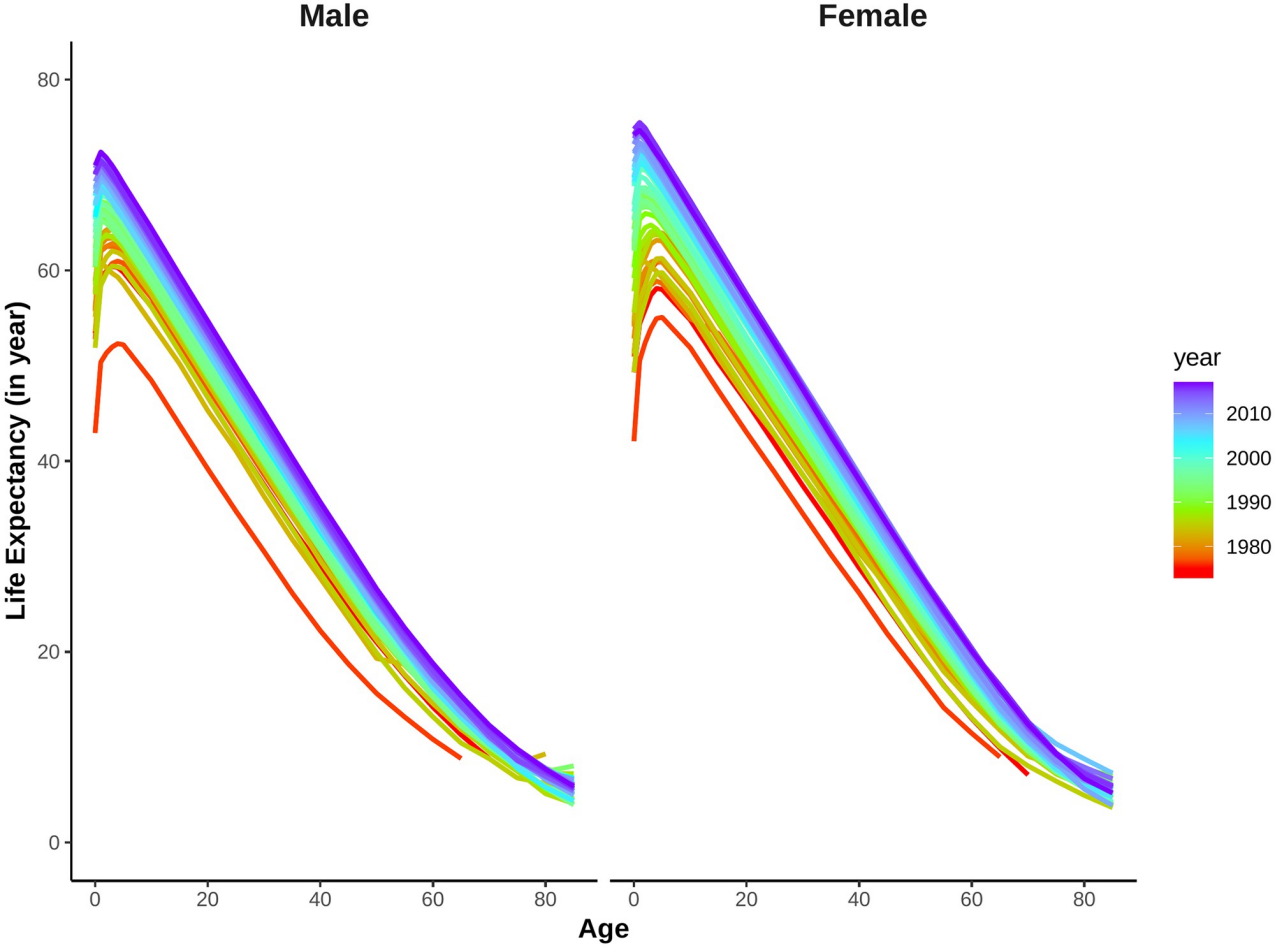
Trend of life expectancies in Bangladesh (Data: Matlab HDSS; 1974–2016).

Fewer application of these methods exist for the populations with higher mortality or passing mortality transition [7, 8]. Structural changes in mortality patterns have occurred during the twentieth century, reducing the relevance of data from the distant past for current forecasts [9]. Presence of early age (infant/child) mortality affects the forecasting; this also restricts most of the models to make forecast for the historical populations from industrialized countries as well [10]. Many of the developing countries still have the problem of notable infant mortality. Due to lack of vital registration system, complete life-tables are unavailable for many developing countries and thus the application of the mortality forecasting techniques was not possible [11]. Bangladesh, a developing country from South Asia, may be considered as an illustrative example on this situation of mortality transition. The country is currently passing through a demographic transition with sharp decline in fertility followed by steady decline in mortality resulting a sharp rise in the life expectancies. The life expectancy at birth for men rose from 52.7 years in 1966 to 71 in 2016, and for women, from 50.7 to 74.2 years [12]. The trend of life expectancy in Bangladesh is sketched in Fig 1.

Two unique characteristics of this transition are reflected in these trend lines for Bangladesh; a rapid fall in infant and child mortality over the time (for both sexes) and a distinct fall in maternal female mortality which is responsible for rise in life expectancies of women between age 20–40 years [12]. Clearly, this change in mortality pattern is not identical for all age groups, the pace of mortality decline in male adult 44 mortality is slower than that of females. Unlike aging societies, the fall in senescence mortality is slower. The decline in early-aged mortality remained as main determinant for rise in life expectancy (Fig 1). Life expectancy at birth is lower than that of age 1 or 2. Similar to many aging populations [13], female life expectancies are higher than that of male counterparts since 1980s. Sharp rise in the life expectancy along with shift in mortality implies upcoming aging in Bangladesh [11]. Due to presence of high fertility till 2000s, however, the effect in change of population structure will not create notable problem in labor market [14]. Most of the previous works analyzed determinants of different age-specific mortality rates in Bangladesh (for example: infant mortality or maternal mortality) rather than ageing related problems or forecasting mortality. Fewer studies exist to explain this mortality transition considering the whole lifespan, whereas nothing exists till now for mortality forecasting. United Nations [15] forecasts are the only source so fart to obtain probabilistic forecast for Bangladesh (for life expectancy at birth only, without considering the entire lifespan).

In this paper we apply the Lee-Carter (LC) method to forecast mortality and life expectancy for Bangladesh. We utilize a functional data analysis approach in LC framework [16] for modeling and forecasting for Bangladesh. Hyndman and Ullah proposed this method to address the problem of lack of a cross-age smoothness [17], heterogeneity of deaths over a long time period and the consideration of only the first principal component in LC variants [16]. Moreover, this model utilizes second- and higher-order principal components to capture additional variation in mortality rates. Previous study obtained better performance of this method for mortality forecasting in comparatively higher mortality regime [8].

## Materials and methods

The following subsection provides details on data used in this study, model fitting, and measures considered for checking forecast accuracy.

### Data

Bangladesh has limited vital registration data as yet. As an alternative, the vital registration and maternal and child health data gathered from Matlab Health and Demographic Surveillance System (HDSS) from Bangladesh is utilized for current study [12]. Since 1966, the Matlab HDSS has maintained the registration of births, deaths, and migrations, in addition to carrying out periodical censuses in Matlab, Bangladesh [12]. Bangladesh became independent in 1971, so data from 1974 to 2016 is considered in current study and life-tables are constructed from midyear population and death counts during this period. The illustration is done all over the paper considering life-tables separately for men and women.

### Model fitting and forecasting

We use standard life-table notations all over the paper. For a period life-table, age-specific mortality rates are defined as,

$$\begin{array}{c} m_{x}=\frac{D_{x}}{P_{x}}, \end{array}$$

where *D*_*x*_ is the observed death counts in a calendar year and *P*_*x*_ is the mid-year population of that year for age group *x*. Typically, deaths are observed at single years of age or in 5-years of age. We reconstruct the life-tables by taking the death counts and population size from Matlab HDSS. The raw data were grouped in 5-years age groups (see S1 Appendix for details).

We apply nonparametric method of mortality forecasting proposed by Hyndman and Ullah [16]. This method is essentially an extension of Lee-Carter (LC) method, proposed to overcome limitations of the LC variants. To explain the method, two factor LC model is,

$$\begin{array}{c} \text{ln}\left(m_{x,t}\right)=a\left(x\right)+b\left(x\right)k_{t}+\epsilon_{t}\left(x\right). \end{array}$$ (1)

Here *m*_*x*,*t*_ is the central mortality rate at age *x* for year *t*; *a*(*x*) is the average of log-mortality at age *x* over time; *b*(*x*) is the first principal component capturing relative change in the log-mortality rate at each age *x*; *k*_*t*_ is the overall level of mortality in year *t* and *ϵ*_*t*_(*x*) is homoskedastic centered error terms. The parameters are subject to two constraints: invariant *a*(*x*) and *b*(*x*) over time and,

$$\begin{array}{c} \sum_{x=0}^{x=p}b\left(x\right)=1\;\text{and},\;\sum_{t=1}^{t=n}k_{t}=0. \end{array}$$

After obtaining the mean log-mortality rates, $\hat{a}\left(x\right)$; singular value decomposition (SVD) is done on $Z_{x,t}=\left[\text{ln}\left(m_{x,t}\right)-\hat{a}\left(x\right)\right]$ to obtain the OLS estimate of LC model. Lee and Carter considered the rank-1 approximation only as it explains most of the variance [6]. If we consider more than one principal components, Eq (1) can be rewritten as,

$$\begin{array}{c} \text{ln}\left(m_{x,t}\right)=a\left(x\right)+b_{1}\left(x\right)k_{1,t}+b_{2}\left(x\right)k_{2,t}+b_{3}\left(x\right)k_{3,t}+\ldots +\epsilon_{t}\left(x\right). \end{array}$$ (2)

To consider these higher order principal components, Hyndman and Ullah combined ideas from functional data analysis, nonparametric smoothing and robust statistics [16]. This technique uses a penalized regression spline with a partial monotonic constraint to smooth the log-mortality rates first. The following continuous smooth function *f*_*t*_(*x*) is assumed for discrete ages,

$$\begin{array}{c} \text{ln}\left(m_{x,t}\right)=f_{t}\left(x_{i}\right)+\sigma_{t}\left(x_{i}\right)\epsilon_{t,i};\;i=1,\ldots ,p;\;t=1,\ldots ,n; \end{array}$$ (3)

where *σ*_*t*_(*x*_*i*_) is the noise component and *ϵ*_*t*,*i*_ is an i.i.d. standard normal variable. Hyndman and Ullah proposed to use weighted penalized regression splines to estimate *f*_*t*_(*x*) [16]. This weighting controls heterogeneity due to *σ*_*t*_(*x*), and a monotonic constraint for upper ages can lead to better estimates. Following Hyndman and Shang, we apply equal weights to the approximate inverse variances, *w*_*x*,*t*_ = *m*_*x*,*t*_*E*_*x*,*t*_, where *E*_*x*,*t*_ represent the population exposed to death at age *x* in time *t* [18]. We use weighted penalized regression splines to estimate the curve *f*_*t*_(*x*) for each year [16]. Weighted penalized regression splines are preferable in terms of computational time and allow monotonicity constraints [16]. Smoothing by splines is important in context of mortality data of Bangladesh; this allows us to construct single-year life-tables for mortality modeling instead of using abridged life-tables.

After smoothing the mortality rates, functional principal component analysis utilizes a set of continuous functions and is decomposed into functional principal components and their associated scores, symbolically,

$$\begin{array}{c} f_{t}\left(x\right)=a\left(x\right)+\sum_{j=1}^{J}b_{j}\left(x\right)k_{t,j}+e_{t}\left(x\right);\;t=1,\ldots ,n. \end{array}$$ (4)

Here *a*(*x*) is the mean function $\left(=\frac{1}{n}\sum_{t=1}^{n}f_{t}\left(x\right)\right)$; *b*_*j*_(*x*) is the set of first *J* functional principal components; *k*_*t*,*j*_ is the set of uncorrelated principal component scores and *e*_*t*_(*x*) is the residual function. It should be noted that *J* < *n* is considered for optimal number of functional principal components. Clearly, Eq (4) is an extension of Eq (1) in case of smoothed mortality rates. We apply the weighted version of Hyndman and Ullah method, where recent years get more weight during model fitting than years from the distant past [16]. The new method can be showed symbolically as follows,

$$\begin{array}{c} f_{t}\left(x\right)={\hat{a}}^{*}\left(x\right)+\sum_{j=1}^{J}b_{j}^{*}\left(x\right)k_{t,j}+e_{t}\left(x\right); \end{array}$$ (5)

where, a*(*x*) is the weighted functional mean, such as,

$$\begin{array}{c} {\hat{a}}^{*}\left(x\right)=\sum_{t=1}^{n}w_{t}f_{t}\left(x\right),\;\sum_{t=1}^{n}w_{t}=1,\;\text{where},\;w_{t}=\kappa {\left(1-\kappa \right)}^{n-t};\;t=1,\ldots ,n. \end{array}$$

This *w*_*t*_ is the new weight defined for 0 < *κ* < 1; a geometrically decaying weight parameter [16]. The optimal value of *κ* is chosen by minimizing an overall forecast error measure within the validation data set among a set of possible candidates [18]. The ARIMA model is suggested to forecast principal component scores, as they have minimum AIC (Akaike information criterion) of the fitted model, however, almost every suitable time series can be applied [19]. Following previous works, we use ARIMA (0,1,0) for mortality forecasting [20].

### Forecast accuracy

We consider the following two measures for checking the forecast accuracy of mortality rates:

mean absolute forecast error,

$$\begin{array}{c} \text{MAE}=\frac{1}{(p+1)q}\sum_{r=1}^{q}\sum_{x=0}^{p}|y_{x,r}-{\hat{y}}_{x,r|r-h}|; \end{array}$$ (6)

mean squared forecast error,

$$\begin{array}{c} \text{MSE}=\frac{1}{(p+1)q}\sum_{r=1}^{q}\sum_{x=0}^{p}(y_{x,r}-{\hat{y}}_{x,r|r-h}{)}^{2}; \end{array}$$ (7)

and for life expectancy at birth, we consider the mean error,

$$\begin{array}{c} \text{ME}=\frac{1}{q}\sum_{r=1}^{q}({\hat{e}}_{0,r}-e_{0,r}). \end{array}$$ (8)

Here *y*_*x*,*r*_ represents the observed mortality rate for age *x* (with highest value of *p* years) in year *r* (represents calendar-years for fitting the model (with highest value of *q* years) and ${\hat{y}}_{x,r}$ represents the forecast; *e*_0,*r*_ represents the observed life expectancy at birth in year *r* and ${\hat{e}}_{0,r}$ represents the forecast. From the available mortality data, we used the last 10 years as the period for forecasting and the previous years as the fitting period. Using the data in the fitting period, we made ten-step-ahead forecasts, and determined the forecast accuracy by comparing the forecasts with the observed data in the hold-out period. All the analysis in this study is performed using R.

## Results and discussion

### Model-fitting

**Fig 2:**
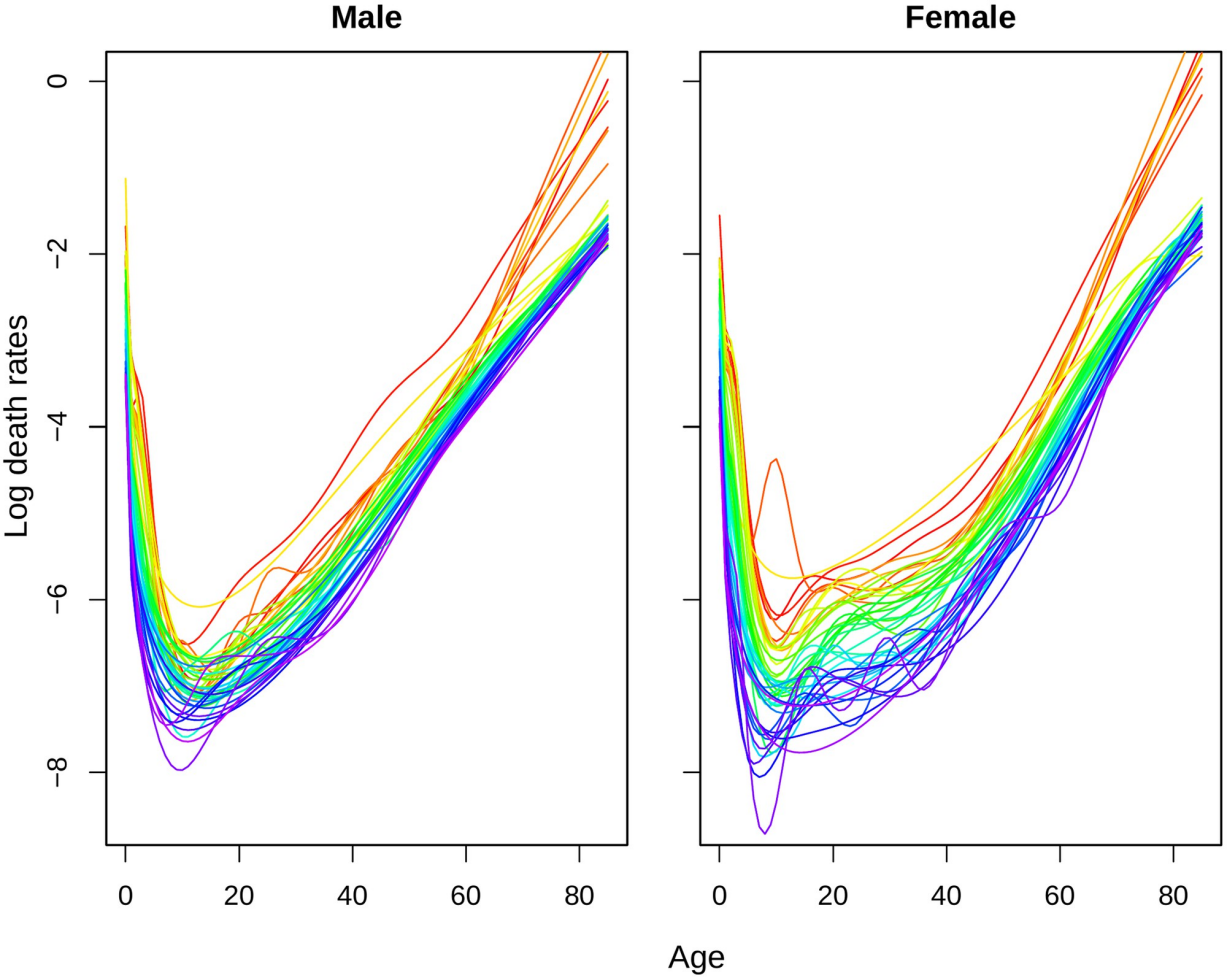
Smoothed log-mortality rates for Matlab HDSS (1974–2016). Years are plotted using a rainbow palette so the earlier years are shown in red, followed by orange, yellow, green, blue and indigo with the most recent years plotted in violet.

In this subsection, we show the findings from the smoothing techniques and estimation of parameters in the fitted model. As we smooth the mortality rates data first, the smoothed mortality rates are illustrated in Fig 2. The transformed errors for smoothing is attached in the S1 Appendix.

The life-tables were constructed considering up to age 85 since 1980s but older life-tables have shorter lifespan [12]. Smoothing by spline are useful in this sense as it reduces loss of information during model fitting for forecasting. Following Hyndman and Ullah, these splines are constrained to ensure that the resulting *f*_*t*_(*x*) is monotonically increasing for *x* > *c* for some *c* (for example 65 years) [16]. Retirement age for Bangladesh is 59 years in most of the places [21], so we consider 60 years for this constraint. Due to life-tables with shorter lifespan in earlier data, the mortality rates for both sexes have a sharper increase in those years after age 60. Besides various causes of deaths, other fluctuations in mortality rates are also visible even after smoothing. An age-sex specific decomposition of the observed mortality rates can explain most of the irregularities during this transition [11].

**Fig 3:**
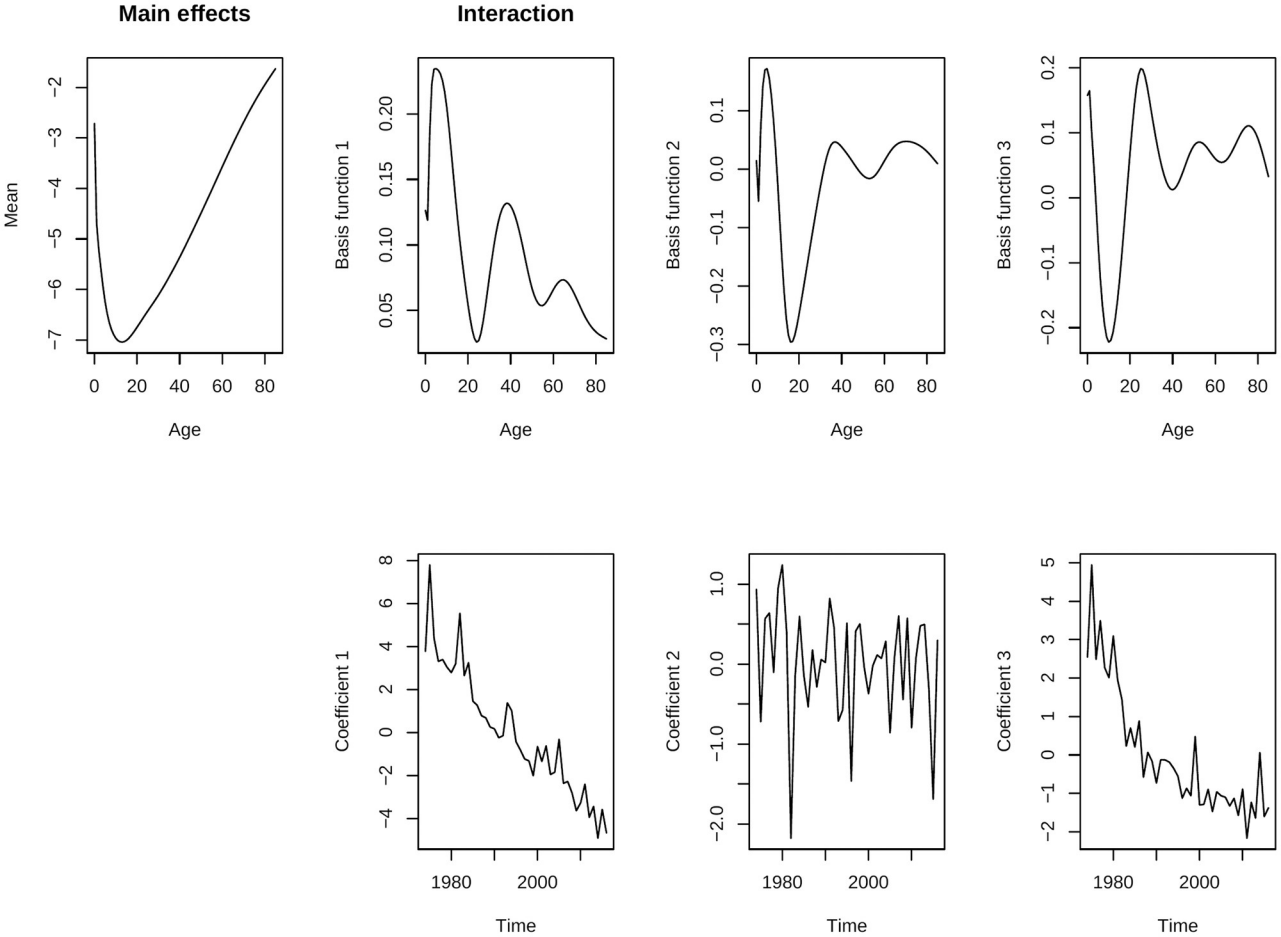
Fitted parameters of the FDA model for men of Matlab HDSS (1974–2016).

**Fig 4:**
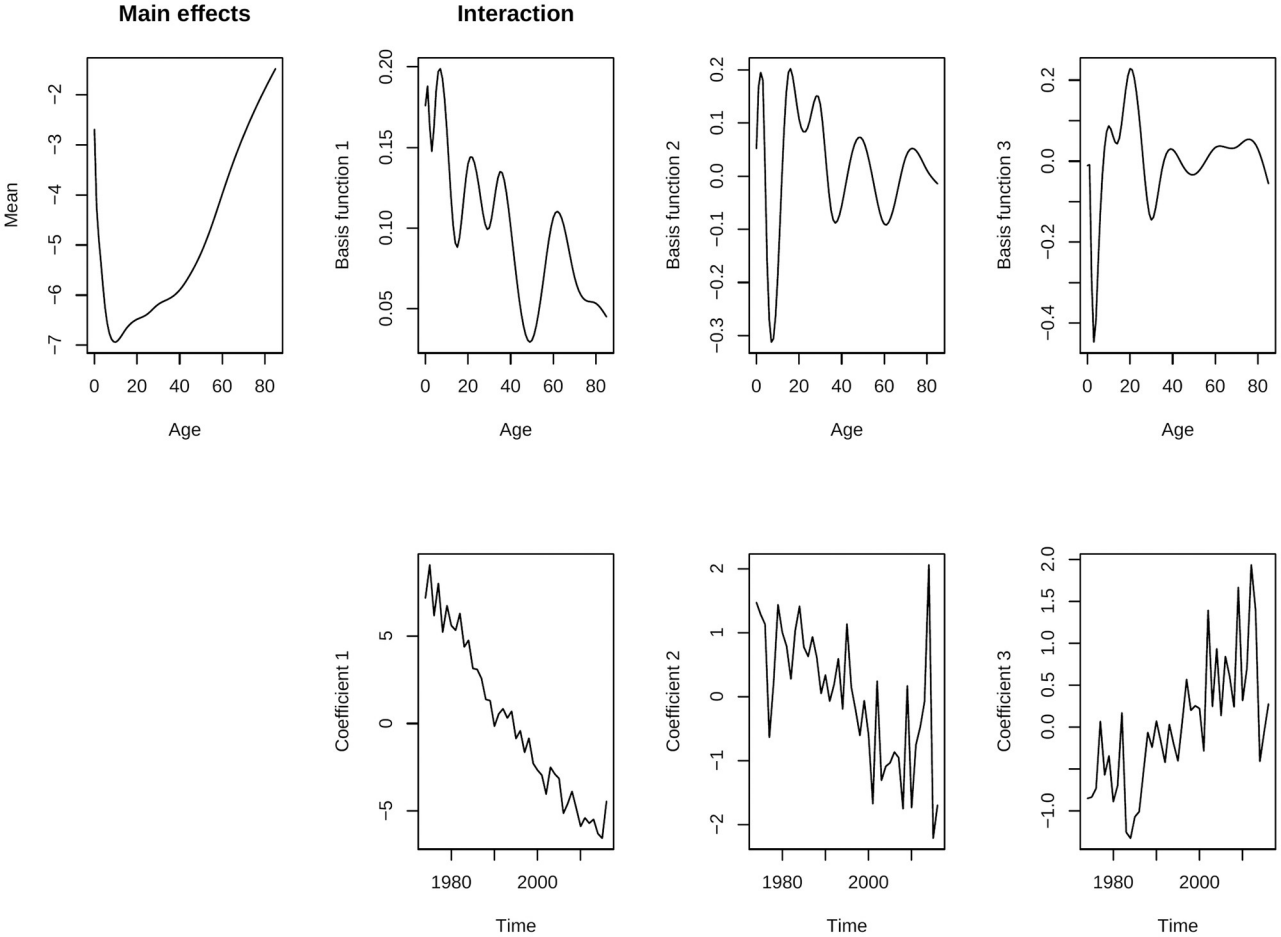
Fitted parameters of the FDA model for women of Matlab HDSS (1974–2016).

After smoothing the mortality rates, we apply the functional data analysis (FDA) method to fit a three component LC model. The variance explained by the fitted FDA models from each of these components are given in Table 1 whereas the fitted parameters are presented in Figs 3 and 4 respectively for men and women of Matlab HDSS.

**Table 1:** Variation explained by the fitted FDA models for Matlab HDSS (1974–2016).

	Variation explained			
Model	*b*_1_	*b*_2_	*b*_3_	Total
Male	85.4%	8.2%	2.5%	96.1%
Female	88.2%	5.9%	2.0%	96.1%

As mentioned before, the FDA method is essentially an extension of the LC method except for number of principal components and smoothing prior to model-fitting. LC model can explain almost 95% variation for most of the low mortality countries For post world war period, additional PCs increase the goodness-of-fit of the model [4, 9]. For Matlab HDSS, the LC model can explain lower than that; only 85.4% variations for men and 88.2% for women for smoothed mortality rates (considering only the first PC in Table 1). This also affects the forecasts, the forecast from LC method shows a big jump-off error and unrealistic forecast for Matlab HDSS. However, one should keep in mind that this LC is fitted over smoothed mortality rates in single year life-tables. The result will be worse for the real data which comes from abridge life-table and moreover the original LC method does not consider any smoothing technique for model fitting [17].

**Fig 5:**
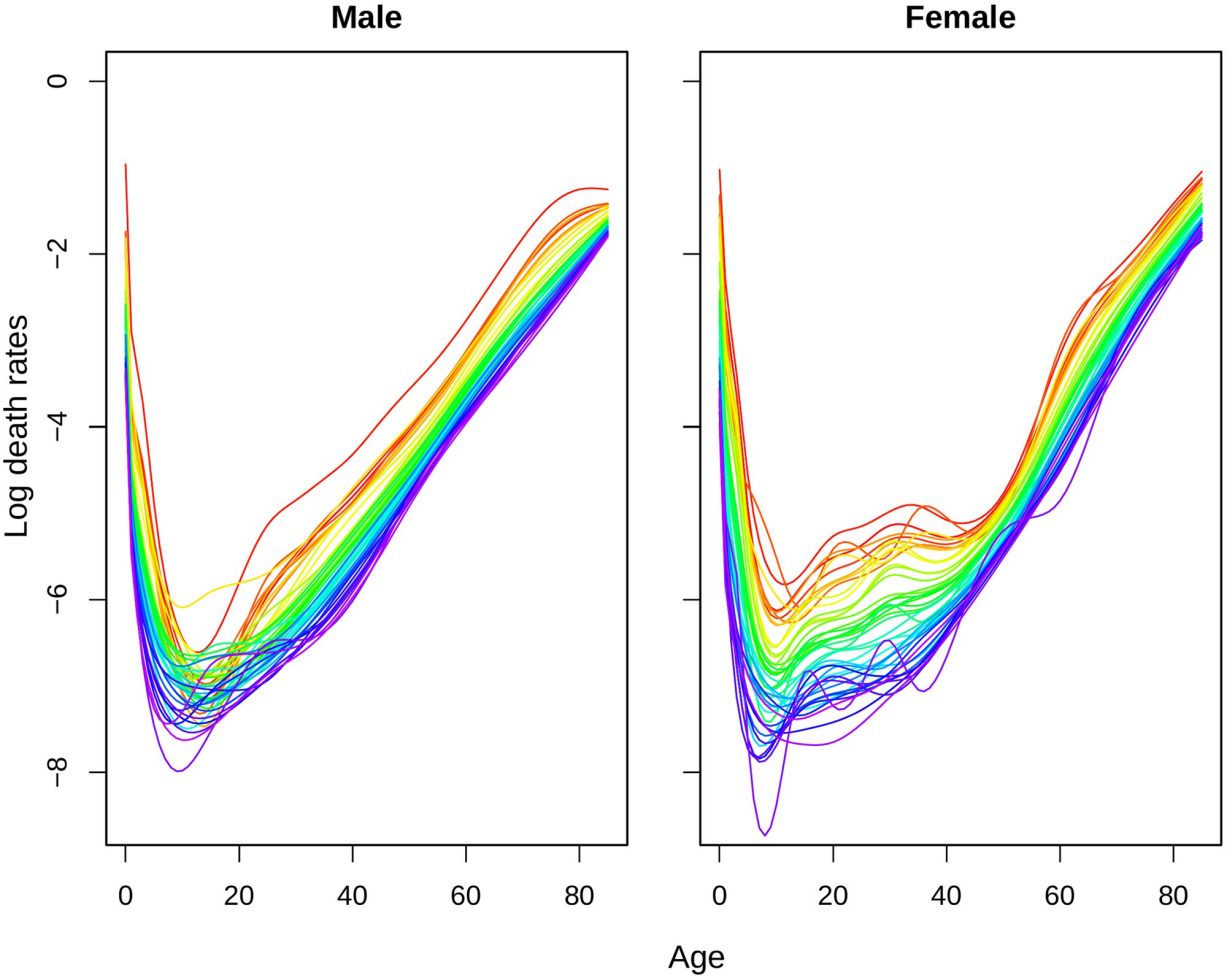
Fitted log-mortality rates from FDA model for Matlab HDSS (1974–2016). Years are plotted using a rainbow palette as before.

The first PC in FDA model (showed using *Basis function 1* in Figs 3 and 4) is just the same as *b*(*x*) of LC model obtained from smoothed mortality rates. This component shows a decline in mortality over time which is usually fastest at childhood and childbearing ages. The second PC models an increase and then decrease explaining 3% of mortality for low mortality countries [4]; for Matlab HDSS it is much higher (Table 1). A large decrease took place at ages around 10 for women whereas for men it happens around age 20. The third PC has very little effect (almost 1% in empirical analysis for low mortality countries) and it explains the change in mortality in older ages where *b*(*x*) = 0. For Matlab HDSS, the impact of the third PC is 2.5% for men and 2% for women. Nevertheless, data quality in the older age groups are doubtful in most of the cases [22]. Together with three PCs, both of the models can explain 96.1% of the observed variation. The fitted mortality rates for Matlab HDSS are given in Fig 5 for the fitting periods. Fitted mortality surfaces and distribution of deaths from reconstructed life-tables from fitted mortality rates are attached in the S1 Appendix.

### Forecast accuracy and point forecast of mortality rates and life expectancy

As mentioned in before in the methodology, we determined the forecast accuracy by comparing the forecasts with the observed data in the hold-out period. The results are given below in Table 2 for Matlab HDSS. The errors are higher for women than that of men. However, the mean forecast errors in life expectancy at birth are positive, which is better from the Actuarial point of view [19].

**Table 2:** Forecast accuracy of FDA method during hold-out period (2007–2016).

Model	MAE(*m*_*x*_)	MSE(*m*_*x*_)	ME(*e*_0_)
Men	0.237	0.125	0.711
Women	0.314	0.179	1.964

**Fig 6:**
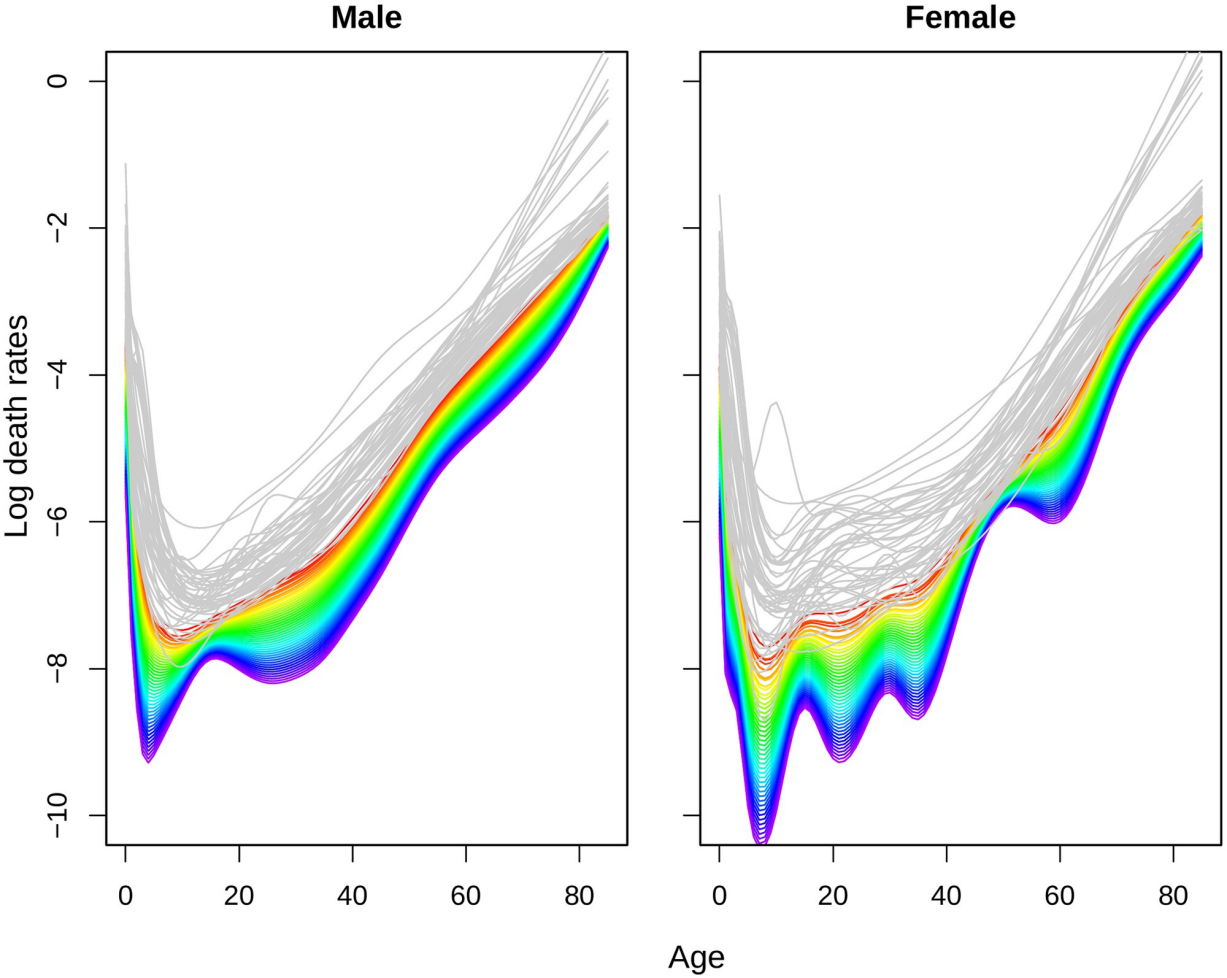
Forecast of log-mortality rates from FDA models for Matlab HDSS (2017–2060). Years are plotted using a rainbow palette as before. Observed mortality rates are showed in gray lines for reference.

The forecast of mortality rates for Matlab HDSS are given in Fig 6 for the period 2017–2060. We plot the observed mortality rates in gray line as well to show the continuation of current mortality improvements over the next four decades. For both sexes the continuation of mortality improvement continued, however, improvement for women are more than that of men. Future mortality rates for women shows rapid fall in future mortality around the age 20, 35, and 65 years. The trend of mortality rates in recent years showed rapid decline around ages 20–40 and older ages for women (after 1980s), which is reflected in the forecast [11].

The forecast of life expectancy at birth and age 60 years are summarized in Table 3 along with last observed life expectancies at those ages. The result shows a sharp rise in life expectancy for both ages. Like most of the ageing societies, the pace of increase is faster for women than that of men [10]. The forecast of mortality rates and life expectancy in a traditional LC setup (without any smoothing and considering first PC only) is included in the S1 Appendix.

**Table 3:** Observed and forecast of life expectancies from FDA method for Matlab HDSS.

Life expectancy (*e*_*x*_)	Male (2016)	Male (2060)	Female(2016)	Female (2060)
*e*_0_	71	82.901	74.2	84.625
*e*_60_	18.8	25.677	20.1	26.584

**Fig 7:**
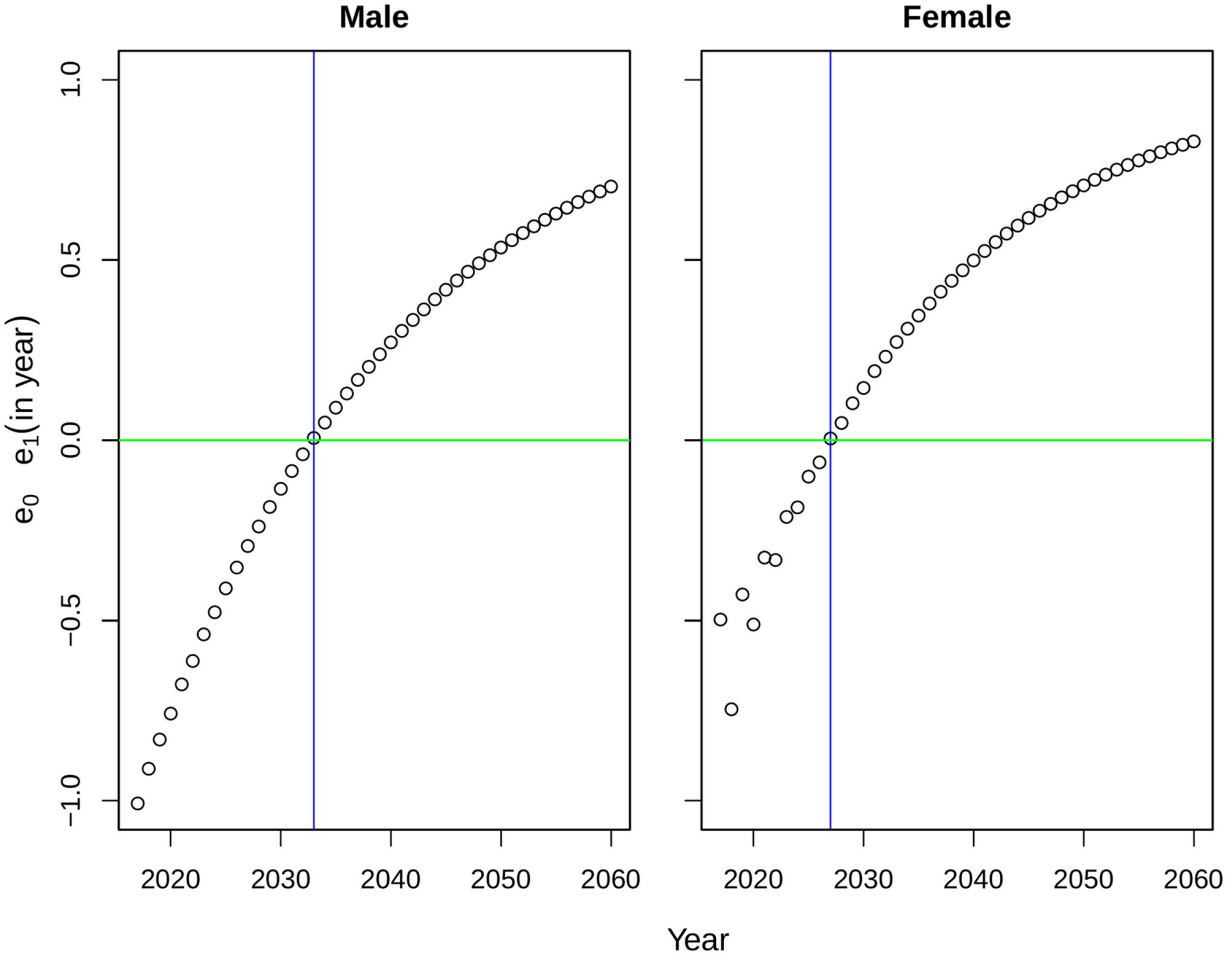
Difference between forecast of *e*_0_ and *e*_1_ for Matlab HDSS (2017–2060). The green line represents null difference after which *e*_0_ will be larger than *e*_1_. The vertical blue line is drawn to identify the possible timing for crossover.

Like many other developing countries, Bangladesh also has the problem of high infant and child mortality [12]. Although it reduced sharply over the years, still the life expectancy at birth is lower than age 1 or 2 years (Figs 1 and 2). Canudas-Romo and Becker quantified the effect of infant mortality on life expectancy at birth and implied that the effect of infant mortality will be minimized when the life expectancy at birth and age 1 will be same [23]. From the obtained forecast of *e*_0_ and *e*_1_, we plot the difference of *e*_0_ and *e*_1_ in Fig 7. From the obtained difference across time, life expectancy at birth will be slightly larger than life expectancy at age 1 from 2033 for men and 2027 for women. Although the difference increased almost linearly after the threshold level, the preliminary fluctuation observed for women are subject to analyze. Measures of forecast accuracy and a brief comparison of forecast with low mortality countries are attached in S1 Appendix.

### Interval forecast of life expectancies

**Fig 8:**
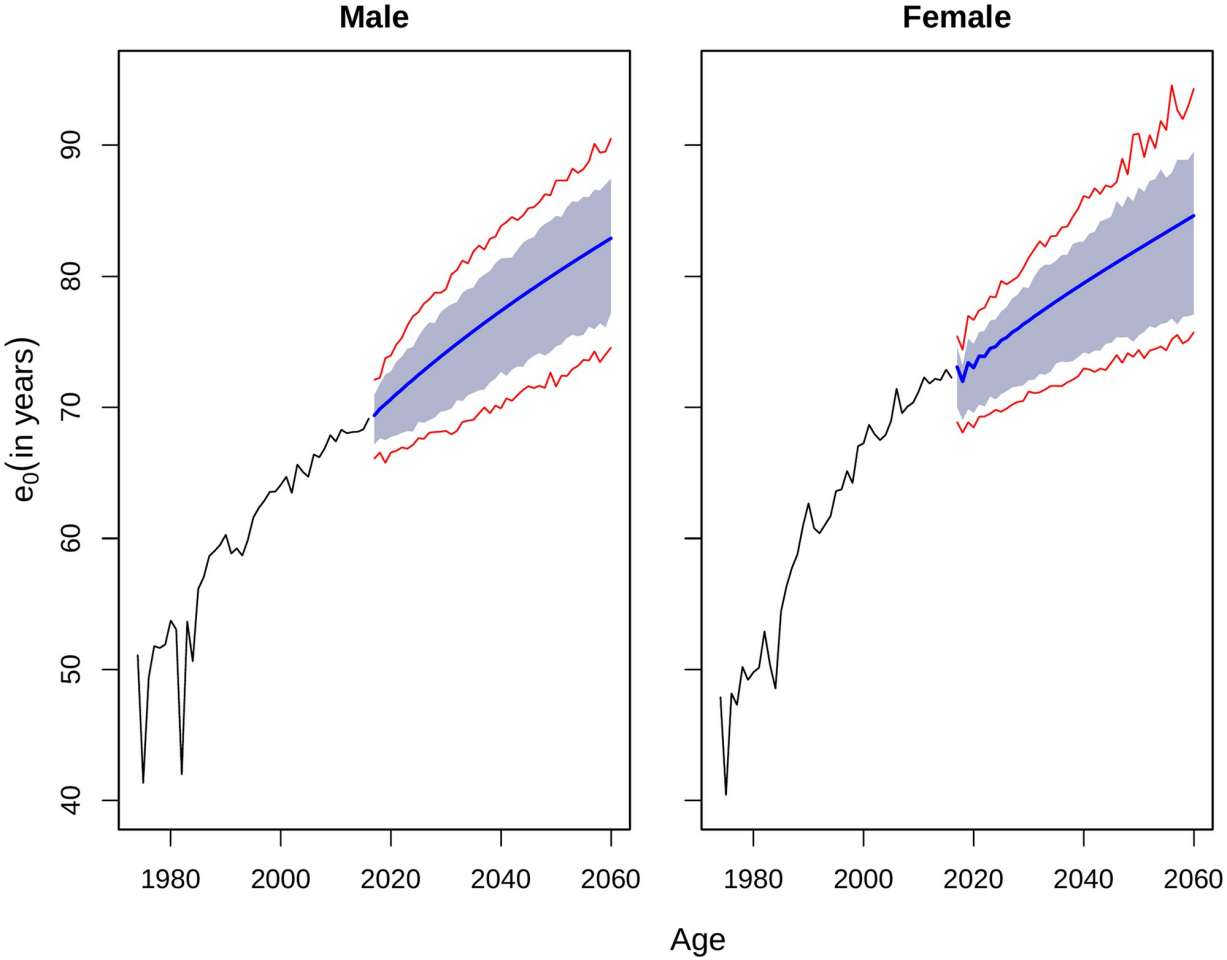
Prediction interval of *e*_0_ by HU, until 2060 for Matlab HDSS. The blue area represents the 80% prediction interval and the red lines indicate the 95% prediction interval.

**Fig 9:**
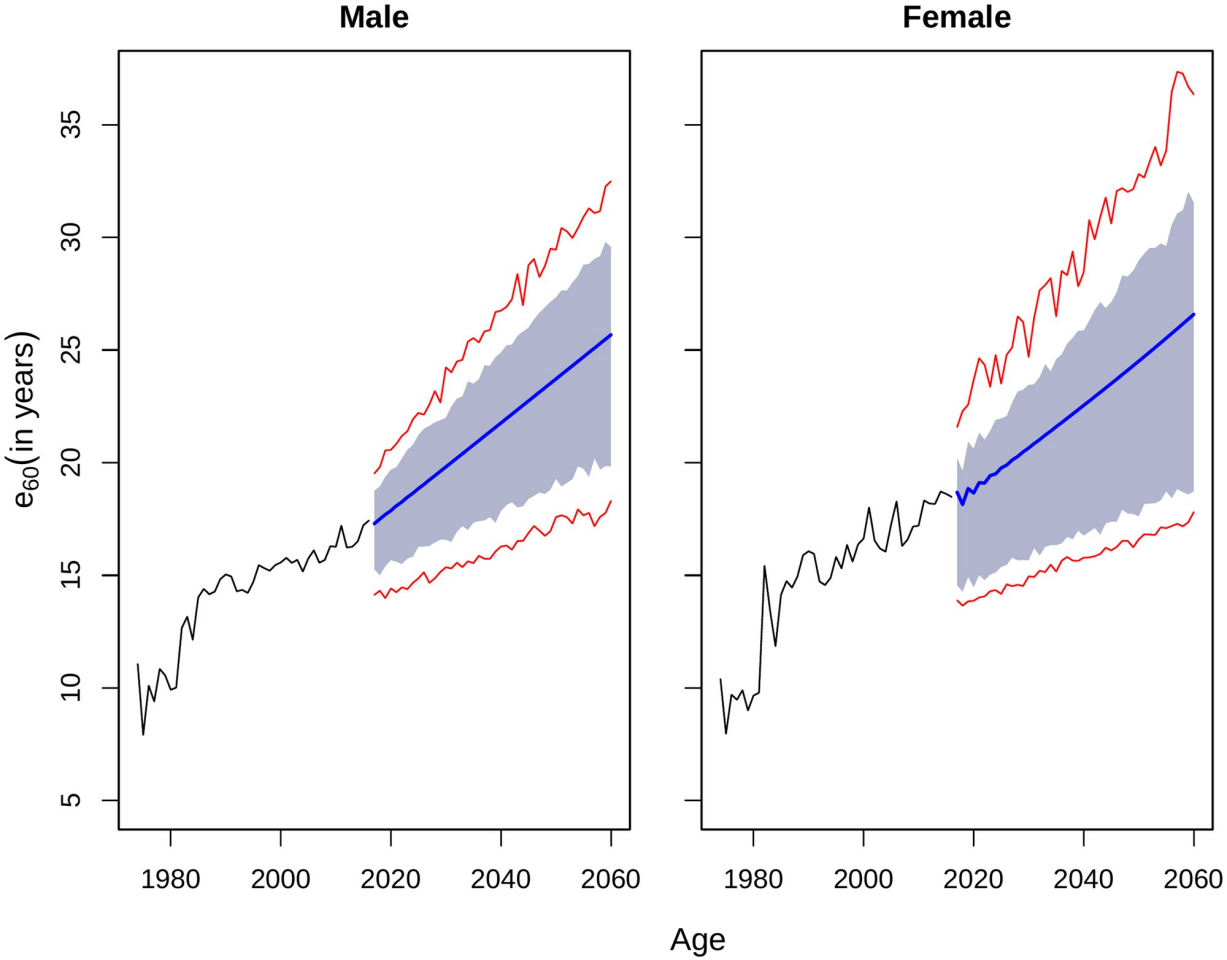
Prediction interval of *e*_60_ by HU, until 2060 for Matlab HDSS. The blue area represents the 80% prediction interval and the red lines indicate the 95% prediction interval.

To construct prediction interval of forecast of life expectancy at birth, we followed the procedure employed by [4]. In this procedure, the fitted mortality rates from forecasting technique is simulated a large number of times (lets say, 500 times) to add disturbance to the time component of the model. Life expectancies are then calculated for each set of the simulated log-mortality rates. Prediction intervals are then constructed by 80% or 95% percentiles of the simulated sets of the life expectancies. The prediction intervals for life expectancy at birth and age 60 are illustrated in Figs 8 and 9 respectively.

LC types of model has an old criticism for producing narrow prediction interval due to lower variance of the estimated parameters [24]. Narrow confidence interval may lead to underestimating the coverage probability. The FDA method could make it worse due to application of smoothing, because smoothing may also reduce the variance in the fitted models [17]. As an alternative solution to overcome that, the second and third PCs add more variance in the model compare to that of original LC model [9]. The difference between upper and lower limits for 95% prediction intervals for life expectancy at birth ends up at 15.95 years for men and 18.59 years for women in Matlab HDSS.

Previous studies also mentioned that the prediction intervals obtained from the LC-type models were too narrow [6, 19]. To check the accuracy of the prediction intervals, we estimated the coverage probability deviance for all the models [19]. This index represents the absolute difference between 0.8 (the nominal coverage probability) and the empirical coverage probability (the actual proportion of the out-of-sample data that falls within the estimated prediction intervals). For a nominal coverage probability of 0.8, the deviance can vary between 0.0 to 0.8. A lower value of the coverage probability indicates a higher accuracy of the prediction intervals for a model. The results are presented in Table 4 along with mean width of prediction interval during the out-of-sample period (2007:2016). Interval forecast for life expectancy at birth were highly underestimated for the fitted model. For both male and female, almost all the years in forecast horizon were underestimated in case of life expectancy at birth. However, the fitted model worked exceptionally well for later age (60 years). The coverage was maximum for both male and females for life4 expectancy at age 60 years as all the forecasted values fall within the interval forecast.

**Table 4:** Coverage probability deviance and mean width of confidence interval (CI) during out-of-sample period (2007:2016) from FDA method for Matlab HDSS.

	Male		Female	
Life expectancy (*e*_*x*_)	Coverage probability	Mean CI (in years)	Coverage probability	Mean CI (in years)
*e*_0_	0.8	13.39	0.6	12.96
*e*_60_	0.0	8.91	0.0	14.24

## Conclusion

In this paper we applied the Lee-Carter method to forecast mortality rates and life expectancy for Bangladesh. We used the functional data analysis of [16] for modeling and forecasting for Bangladesh which is an extended version of the LC method with more than one principal component and nonparametric smoothing. The modeling of mortality rates were performed over smoothed mortality rates to overcome the limitations of original LC method. The long run forecasts show continuation of current trend of mortality improvement for Bangladesh. We also predict about the possible timing for significantly reduced effect of infant mortality in life expectancy at birth for Bangladesh. To best of our knowledge, this study is the first probabilistic approach to forecast mortality rates and life expectancy for Bangladesh (except for current UN forecasts for life expectancy at birth).

The study has several limitations, mainly due to data. Lack of mortality data with good quality limits the scope of many aspects available with the forecast methods. Although Matlab HDSS is recognized as one of the long-term demographic surveillances sites for a developing country, still this data represents only a specific region of Bangladesh only. Lack of detailed mortality data from central vital statistics limits the applicability of the results for policy making in a larger scale. Application of indirect estimation techniques may provide suitable solution for this problem [25]. Another shortcoming of the data is that some unexplainable fluctuations in mortality rates are visible over the lifespan. These irregularities form mortality transition effects the forecast as a consequence (Fig 7). Theoretical burdens were also present in the methodology. All of these mortality forecasting methods were developed mainly for industrialized countries characterized by low mortality, high life expectancies, lower adult and early senescence mortality, a stable pattern of mortality transition over time, and high data quality [26]. Highly irregular trend of the time components (due to mortality transition) affected the forecasts for Bangladesh (Figs 3 and 4). Although the weighted FDA model is defined for giving higher importance in mortality rates in recent years, it is also not free from limitations (see S1 Appendix for an example). Previous studies also mentioned that none of the available mortality forecast methods are perfect for all populations [19].

Several possible extensions of this work might be possible conditional on availability of good quality mortality data. Due to the limitation of assumptions, we could not analyze many other available mortality forecast method or neither did we compare our findings with UN forecasts; although the last one forecasts life expectancy at birth only. Coherent mortality forecasts gained popularity in the last decade [27], it may be highly effective for Bangladesh. Cause-specific life-table will allow further insight of this mortality transition and its reflection in future mortality. Two possible methodological developments can be proposed as well. First, a significant improvement would be to develop a new forecasting technique flexible enough to handle this sort of irregularities in mortality trends. Second, new extrapolation technique will be useful to extend the available mortality data in senescence ages for population passing mortality transition. Nevertheless, a mortality forecast method considering distribution of deaths will be more insightful in terms of accuracy and policy making for Bangladesh.

## Supporting information

S1 Appendix Appendix A–C. (PDF)
