# Supplementary material for: Stochastic mortality forecasts for Bangladesh
Source: PLoS One. 2022 Nov 10;17(11):e0276966. doi: 10.1371/journal.pone.0276966 (PMC9648772; doi:10.1371/journal.pone.0276966)
Supplement: S1 Appendix — (PDF) [file pone.0276966.s001.pdf]

Stochastic Mortality Forecasts for Bangladesh

Ahbab Mohammad Fazle Rabbi[1], Hafiz T. A. Khan[2,3]
1 Department of Population Sciences, University of Dhaka, Bangladesh
2 Professor of Public Health & Statistics, University of West London, United Kingdom
3 Associate Professorial Fellow, The Oxford Institute of Population Ageing, The University of Oxford, United Kingdom

Appendix A: Forecast of mortality rates and life expectancy from Lee-Carter method

For fitting the Lee-Carter model, we use the observed data without any smoothing or extrapolation; so the ages are in 5 years age groups. The model is fitted without any adjustment on the time component (Lee and Carter 1992; Lee and Miller 2001; Booth et al. 2002) due to missing values. The model for men explained only 66.1% of the observed variation while for women it explained 75.8% variation.

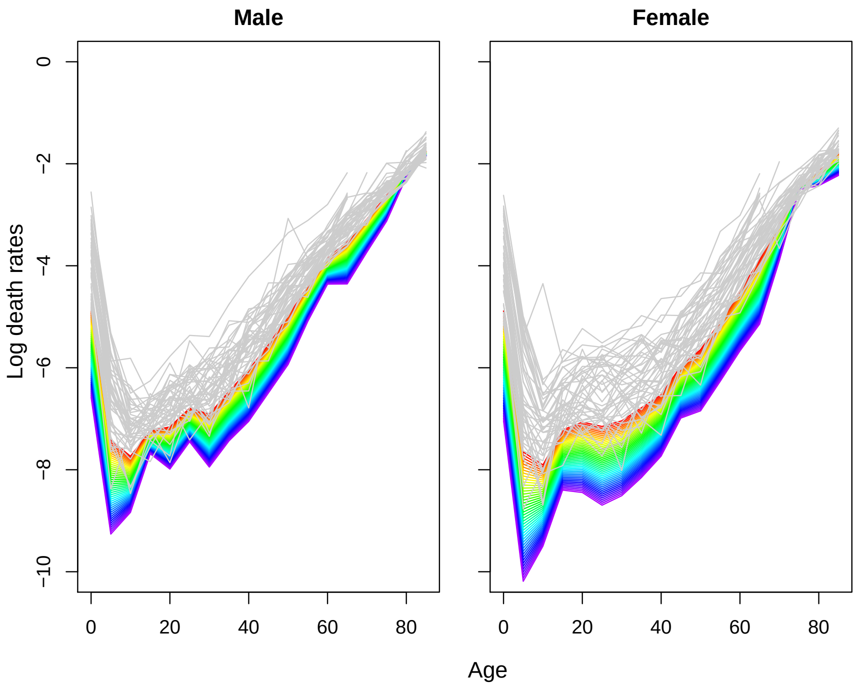

Figure 1: Forecast of log-mortality rates from Lee-Carter method for Matlab HDSS (2017–2060). Years are plotted using a rainbow palette so the earlier years are shown in red, followed by orange, yellow, green, blue and indigo with the most recent years plotted in violet. Observed mortality rates are showed in gray lines for reference to compare the mortality improvements in future.

Table 1: Observed and forecast of life expectancy from Lee-Carter method for Matlab HDSS.

Life expectancy (e_x)	Male (2016)	Male (2060)	Female(2016)	Female (2060)
e_0	71	74.612	74.2	77.596
e_{60}	18.8	21.922	20.1	23.665

Appendix B: Details of model fitting

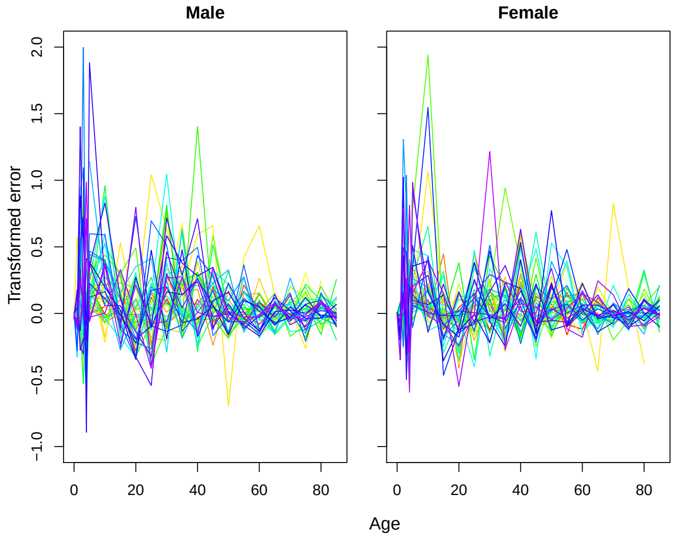

Figure 2: Errors in smoothing for Matlab HDSS during fitting period (1974–2016). Years are plotted using a rainbow palette as before.

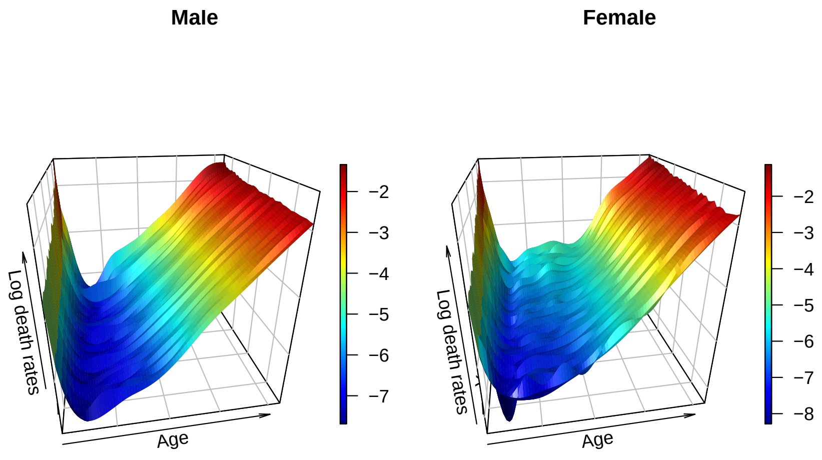

Figure 3: Fitted mortality surfaces from FDA models for Matlab HDSS (1974–2016).

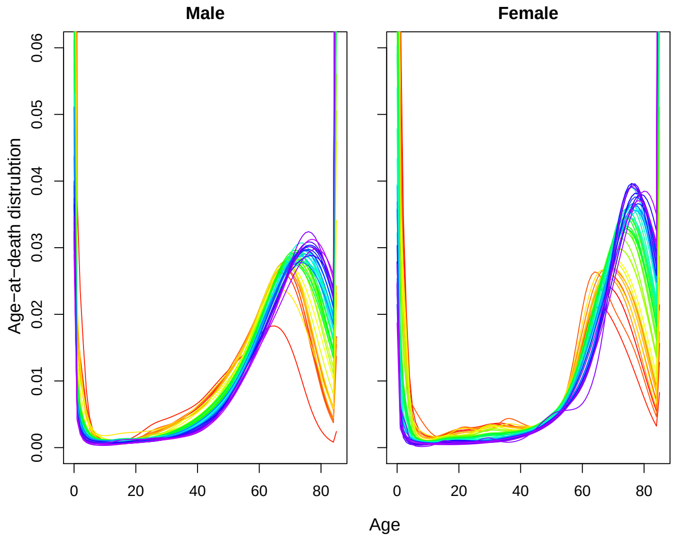

Figure 4: life-table distribution of deaths from from fitted FDA models for Matlab HDSS (1974–2016). The life-tables are reconstructed form fitted mortality rates. Years are plotted using rainbow pallets as before.

Appendix C: Comparison of future longevity of Bangladesh and some low mortality countries

We considered Australia, France, Japan and United States for this comparison. Time series data for a long period are available for all these countries in the Human Mortality database (HMD 2020). For this comparison, we considered only the weighted FDA model and fitting years of 1974–2016 (as we have for Matlab HDSS). All the models are fitted considering life-tables up to age 85 years. Two unusual results may seen in the forecast; a declining trend of future e_0 for women of United States, and lower future life expectancies for Australian women than that of Australian men.

Table 2: Observed and forecast of life expectancy at birth from FDA method for Bangladesh and some low mortality countries at 2060.

Country	Male (2016)	Male (2060)	Female(2016)	Female (2060)
Australia	81.101	90.851	85.269	90.352
Bangladesh	71	74.612	74.2	77.596
Japan	81.348	89.834	88.037	98.173
France	79.539	88.630	85.795	94.370
U.S.A.	76.535	84.956	81.582	65.427

Table 3: Observed and forecast of life expectancy at age 60 from FDA method for Bangladesh and some low mortality countries at 2060.

Country	Male (2016)	Male (2060)	Female(2016)	Female (2060)
Australia	24.307	32.702	27.341	31.697
Bangladesh	18.8	25.677	20.1	26.584
Japan	24.074	31.044	29.854	39.055
France	23.353	30.590	28.047	35.774
U.S.A.	22.197	30.065	25.247	44.412

References

Booth, H., Maindonald, J., and Smith, L. (2002). Applying Lee-Carter under conditions of variable mortality decline. *Population studies*, 56(3):325–336.

HMD (2020). *Human Mortality Database*. University of California, Berkeley (USA), and Max Planck Institute for Demographic Research (Germany).

Lee, R. and Miller, T. (2001). Evaluating the performance of the Lee-Carter method for forecasting mortality. *Demography*, 38(4):537–549.

Lee, R. D. and Carter, L. R. (1992). Modeling and forecasting us mortality. *Journal of the American statistical association*, 87(419):659–671.
